# Emerging roles of lipid and metabolic sensing in the neuroendocrine control of body weight and reproduction

**DOI:** 10.3389/fendo.2024.1454874

**Published:** 2024-09-03

**Authors:** Elvira Rodríguez-Vázquez, Álvaro Aranda-Torrecillas, María López-Sancho, Juan M. Castellano, Manuel Tena-Sempere

**Affiliations:** ^1^ Instituto Maimónides de Investigación Biomédica de Córdoba (IMIBIC), Córdoba, Spain; ^2^ Department of Cell Biology, Physiology and Immunology, University of Córdoba, Córdoba, Spain; ^3^ Hospital Universitario Reina Sofia, Córdoba, Spain; ^4^ CIBER Fisiopatología de la Obesidad y Nutrición (CIBEROBN), Instituto de Salud Carlos III, Córdoba, Spain

**Keywords:** lipid sensing, reproduction, mTOR, AMPK, sirtuins, ceramides, fatty acids, Kiss1

## Abstract

The hypothalamus lies at the intersection of brain and hormonal mechanisms governing essential bodily functions, including metabolic/body weight homeostasis and reproduction. While metabolism and fertility are precisely regulated by independent neuroendocrine axes, these are tightly connected, as reflection of the bidirectional interplay between the energy status of the organisms and their capacity to reproduce; a connection with important pathophysiological implications in disorders affecting these two crucial systems. Beyond the well-characterized roles of key hormones (e.g., leptin, insulin, ghrelin) and neuropeptides (e.g., melanocortins, kisspeptins) in the integral control of metabolism and reproduction, mounting evidence has pointed out a relevant function of cell energy sensors and lipid sensing mechanisms in the hypothalamic control of metabolism, with prominent roles also for metabolic sensors, such as mTOR, AMPK and SIRT1, in the nutritional regulation of key aspects of reproduction, such as pubertal maturation. We provide herein a synoptic overview of these novel regulatory pathways, with a particular focus on their putative function in the metabolic control of puberty, and delineate new avenues for further exploration of the intricate mechanisms whereby metabolism and reproduction are tightly connected.

## 1 Introduction: neuroendocrine mechanisms for the control of body weight and reproduction

Metabolism and reproduction are two closely related bodily functions essential for the survival of the organism and the species, respectively. An unequivocal indication of this interaction is that significant disruptions in energy balance, ranging from malnutrition to obesity, are often associated not only with several metabolic alterations, but also with pubertal disorders and reduced reproductive capacity in adulthood (1, 2). Similarly, certain reproductive disorders, such as hypogonadism, may exacerbate an altered metabolic state, such as that bound to obesity (3).

Although the mechanisms linking energy balance and reproduction are intricate and not fully understood yet, it is known that they primarily have a neuroendocrine basis. This involves the complex interaction between specific peripheral signals, particularly metabolic hormones, and molecules generated by the central nervous system, mainly neurotransmitters and neuropeptides (2, 4). The coordination of this interaction occurs primarily in the hypothalamus, a small brain region located below the thalamus that operates as an integrator hub for these signals and produces appropriate homeostatic responses to support the proper functioning of our organism (5).

Leptin, ghrelin, and insulin are essential hormones regulating energy balance and reproductive function. While these hormones have effects at local and peripheral levels, it has been conclusively documented that their primary influence on energy balance and reproductive function occurs through their actions at the hypothalamic level (2, 4, 6). These hormones are now known to directly or indirectly affect specific groups of neurons that play a key role in controlling metabolism and reproduction, which are mainly located in the hypothalamic arcuate nucleus (ARC) or preoptic areas (2). These include: (i) orexigenic neurons co-expressing neuropeptide Y (NPY) and agouti-related peptide (AgRP) – i.e., NPY/AgRP neurons; (ii) anorexigenic neurons co-expressing the peptide products of proopiomelanocortin (POMC), such as alpha-melanocyte-stimulating hormone (α-MSH), and the cocaine- and amphetamine-regulated transcript (CART) – i.e., POMC/CART neurons; (iii) neurons producing gonadotropin-releasing hormone (GnRH), which are essential for the ultimate activation of reproductive axis at puberty and its regulation in adulthood; and (iv) neurons modulating the activity of GnRH neurons, including prominently neurons producing the neuropeptides kisspeptins – named Kiss1 neurons, which are potent stimulators of the reproductive axis (7).

In addition, neuronal populations at other key hypothalamic nuclei, such as the ventromedial (VMH) and dorsomedial (DMH) hypothalamic nuclei, as well as the lateral hypothalamus, have been shown to participate in the control of feeding, energy balance and thermoregulation (8), as well as their interplay with reproductive hormones, such as estrogens (9). Similarly, the relevance of the suprachiasmatic nucleus (SCN) in the regulation of circadian rhythms modulating reproductive and metabolic function (10), as well as the key role of the ventral premammillary nucleus (PMV), as an important integrating node of metabolic and reproductive status, have been documented (6). Altogether, these heterogeneous neuronal components and the peripheral signals they interact with constitute the main neuroendocrine pathways involved in controlling energy balance and reproduction.

In the same vein, a very recent study, which integrated information from seventeen single-cell RNA sequencing datasets into a mouse hypothalamic cell atlas (named HypoMap), has highlighted the complex heterogeneity of the neuronal populations and subpopulations within the hypothalamus and the existence of differential transcriptional responses to specific conditions of metabolic stress, such as fasting (11). This heterogeneity is not restricted to neuronal populations, but affects also glial cells, including astrocytes, oligodendrocytes and their precursor cells, microglia, ependymal cells and tanycytes, which are highly relevant in the modulation of hypothalamic circuits (12). Indeed, compelling evidence has been presented for the involvement of these glial cells in the control of energy balance, including modulation of leptin responses, nutrient sensing and metabolite transport (12, 13), and more recently in the modulation of the reproductive axis by metabolic cues, via kisspeptin-mediated astrocyte signaling (14). Altogether, these heterogeneous neuronal and glial components illustrate the high degree of complexity and sophistication of the mechanisms involved in controlling energy balance and reproduction.

Notably, while the biological actions of the metabolic hormones and neuropeptides indicated above have been extensively characterized in the last three decades, in recent years, novel molecular mechanisms for the homeostatic control of bodily metabolism and reproduction, operating at the hypothalamus, particularly in some of the above neuronal systems, have been unveiled. Part of these recent developments are summarized here, with a specific focus on new pathways putatively involved in the metabolic control of puberty and fertility.

## 2 Role of energy sensors in the hypothalamic control of energy homeostasis

Energy sensors are molecular and cellular systems that perceive the energetic state of a cell or, when operating in specific cellular pathways, of an organism. A caloric nutrient sensor is typically a protein that identifies a specific macronutrient, prompting a cellular response that alters nutrient distribution, animal feeding behavior and/or its metabolic rate. These sensors can operate intracellularly, detecting nutrient flux within metabolic pathways, or extracellularly, perceiving nutrients in the surrounding environment (15, 16). The intricate network of hormonal and neural pathways linking multiple nutrient-sensing organs —such as the intestine, pancreas, liver, adipose tissue, and the brain— suggests a vast communication among nutrient-sensing cells across diverse organ systems (17). Among others, recent studies have demonstrated that pathways, such as AMPK, mTOR and SIRT1, the latter as prominent member of the sirtuin family, are pivotal elements in adaptive responses to changes in body nutritional status and energy reserves, therefore contributing to modulate the energetic and reproductive status of the organism (18).

### 2.1 AMP-activated protein kinase and the neuroendocrine control of metabolism

AMPK is an ubiquitous, highly-conserved kinase, composed of one catalytic subunit (α) and two regulatory subunits (β and γ) (19). AMPK acts as a *bona fide* cell energy sensor, since its activity is driven by changes in the abundance of adenine nucleotides, i.e., AMP, but also ADP and ATP, within the cells. Thus, conditions of energy deprivation result in enhanced activity of AMPK, which in turn represses ATP-consuming phenomena, while it enhances ATP-producing processes. This leads to the restoration of AMP: ATP and ADP: ATP ratios, therefore contributing to energy homeostasis at the cellular level. Importantly, besides this function, acting in specific hypothalamic circuits, AMPK plays a fundamental role in the maintenance of whole-body energy balance, by promoting feeding and catabolic reactions, whereas it suppresses anabolic pathways systemically (17, 20).

In line with this systemic function, in recent years, AMPK-mediated pathways in the hypothalamus have been shown to operate as the main canonical route whereby multiple endocrine factors actively participate in the precise regulation of whole-body metabolism and weight homeostasis. This pivotal role as metabolic mediator has been documented not only for essential metabolic hormones, such as those coming from the adipose tissue (leptin), gut (ghrelin, glucagon-like peptide-1, GLP-1), gonads (estrogens) and thyroid gland (thyroid hormones), but also metabolites, such as glucose (21). Thus, AMPK activity in the hypothalamus seemingly orchestrates a diversity of responses, including stimulation of food intake and modulation of food preferences, regulation of glucose homeostasis (22), suppression of brown adipose tissue thermogenesis (23), and regulation of hepatic function (23), just to highlight its most prominent functions. Interestingly, according to experimental studies, these activities appear to display a considerable degree of nucleus-specificity within the hypothalamus, so that while the function of AMPK to enhance food intake seems to reside mainly in the ARC, the capacity of AMPK to suppress thermogenesis is located mainly at the VMH (17). While the cellular substrate for these differential actions is yet to be fully characterized, it has been shown that AMPK signaling within ARC POMC and AgRP neurons is essential for feeding control (24) whereas neurons expressing steroidogenic factor 1 (SF1) in the VMH play a crucial role in mediating the thermogenic-regulatory actions of AMPK, via sympathetic regulation of brown adipose tissue activity (25).

### 2.2 The mammalian target of rapamycin and the neuroendocrine control of metabolism

mTOR is an evolutionary conserved factor belonging to the PI3K-related kinase family. It forms two multimeric complexes, mTORC1 and mTORC2, through their respective interactions with integral proteins, RAPTOR and RICTOR (26). Activation of mTORC1 leads to the inactivation of mTORC2 and vice versa; thus, the mTORC1/mTORC2 tandem represents an essential component of a negative feedback loop to ensure proper signaling. At the cellular level, mTORC1 senses and becomes activated by nutrient abundance, by detecting different cues, from amino acids to cell stress and energy status, therefore linking these to cell growth and proliferation (27). Accordingly, mTOR and AMPK operate in a reciprocal manner, so that conditions that activate mTOR suppress AMPK activity, and vice versa (28).

At the systemic level, mTOR signaling in the mediobasal hypothalamus becomes activated by leptin, as a signal of energy abundance (29), and mediates, at least partially, the feeding-promoting effects of ghrelin, as putative signal of energy deficit (30), thereby coordinating appropriate feeding and metabolic responses (31). In addition, disruption of mTORC2 in neurons led to increased fat composition, adiposity, and impaired glucose tolerance, while deletion of RICTOR in POMC neurons resulted in obesity, hyperphagia, and glucose intolerance (32), therefore supporting a relevant role of central mTOR signaling in the integral control of metabolism. Of note, as it is the case also for AMPK, the hypothalamic actions of mTOR seem to display some degree of nucleus- and cell-specificity, as demonstrated by the fact that mTOR activity in the ARC and VMH was oppositely regulated by fasting and leptin deficiency (33). These findings, together with the interplay of brain mTOR with other metabolic hormones, such as nesfatin-1 (34), illustrate the complexity of the mode of action of mTOR-dependent pathways in the central control of metabolism.

### 2.3 SIRT1 and the neuroendocrine control of metabolism

Sirtuins are a family of regulatory proteins, highly conserved across evolution, whose initial member is Sir2, identified in *Saccharomyces cerevisiae* (35). In mammals, up to seven sirtuins, including nuclear and mitochondrial proteins, have been identified; SIRT1 being the most prominent and best characterized member of the sirtuin superfamiliy (36). From a functional standpoint, SIRT1 acts as a NAD^+^-dependent deacetylase (37), with capacity to erase acetylation marks from histones and other protein targets. Thereby, SIRT1 can epigenetically modulate multiple biological processes, seemingly including lifespan and healthy ageing (35). Notably, SIRT1, as well as other sirtuins, operate as a genuine cell energy sensor, since their activation is dictated by fluctuations in the cellular levels of the cofactor, NAD^+^, and its related intermediary products, NADH and nicotinamide. Thus, conditions of energy deprivation, such as caloric restriction, that enhance NAD^+^/NADH and NAD^+^/nicotinamide ratios, are known to cause accumulation and activation of SIRT1 in different tissues, including the brain.

Indeed, while SIRT1 is expressed in multiple peripheral tissues, where it is involved in different metabolic adaptive responses in the pancreas, muscle, liver and adipose tissue, SIRT1 is known to be expressed also in the CNS, where it has been shown to participate in the regulation of systemic homeostatic responses, including food intake and energy expenditure (38). In fact, in adulthood, high expression levels of SIRT1 have been reported in different hypothalamic nuclei, including the VMH, dorsomedial and paraventricular nuclei, as well as the ARC (39). On the latter, SIRT1 has been shown to be expressed and operate in essential neuronal populations in the control of metabolic homeostasis, such as POMC and AgRP neurons (38, 40). Moreover, hypothalamic SIRT1 signaling has been shown to participate in conveying the effects of key metabolic hormones, such as ghrelin (41), while SIRT1 in POMC neurons mediates, at least partially, the effects of leptin on PI3K signaling in this neuronal population and leptin-induced remodeling of white adipose tissue (42). Of note, SIRT1 protein levels are elevated in the hypothalamus in conditions of energy deprivation (39); a profile that is mirrored by hypothalamic AMPK levels, which are also elevated under energy deficit. In fact, SIRT1 and AMPK are both fuel-sensing molecules that reciprocally activate each other, therefore contributing to mediate metabolic adaptations to conditions such as energy deprivation (43). Importantly, the hypothalamic actions of SIRT1 are not restricted to ARC neuronal populations; thus, ablation of SIRT1 in SF1 neurons, abundantly expressed in the VMH, is bound to metabolic perturbations, while over-expression of SIRT1 in this neuronal population protects from diet-induced obesity and insulin resistance (44). In addition, other sirtuins, such as SIRT3 and SIRT6, have been shown to operate at the hypothalamic, and particularly in POMC neurons, to contribute to energy homeostasis and adaptive responses to lessen the metabolic impact of obesity (38, 45, 46).

## 3 Role of energy sensors in the hypothalamic control of reproduction

Hypothalamic circuits engaging cellular energy sensors, such as AMPK, mTOR and SIRT1, have been shown also to participate in the metabolic regulation of the neuronal pathways governing puberty onset and the reproductive axis. Indeed, while GnRH neurons in the basal forebrain operate as the main output pathway for the brain control of puberty and fertility, the secretory activity of GnRH neurons is exquisitely dependent on the regulatory actions of upstream regulatory circuits, including prominently Kiss1 neurons, producing kisspeptins, which participate in conveying the modulatory effects of metabolic cues on GnRH neurons. Accordingly, the activity of these sensors has been shown to regulate, either directly or indirectly these key neuronal populations, as a means to funnel the influence of nutritional and metabolic signals to the centers governing the reproductive axis.

### 3.1 AMPK and the metabolic control of puberty and reproduction

The potential function of AMPK in the regulation of the reproductive axis by metabolic cues has been explored in recent years, with a particular focus on its role in the modulation of Kiss1 and GnRH neurons. In line with the capacity of conditions energy deficit to enhance AMPK activity in the hypothalamus, pharmacological activation of brain AMPK has been shown to impair ovarian cyclicity (47) and substantially delayed puberty onset in female rats (48). Initial fragmentary evidence suggested that this effect might involve an inhibition of the Kiss1 system since the adipose hormone, adiponectin, was shown to suppress *Kiss1* expression via induction of AMPK activity in the GnRH cell line, GT1-7 (49). This possibility was later documented by a body of experimental evidence from genetically-modified mouse lines. Thus, congenital ablation of the AMPKα2 subunit from Kiss1-expressing cells resulted in resilience to the inhibitory impact of fasting (50), supporting the view that AMPK activity in Kiss1 neurons conveys a negative influence in conditions of food deprivation bound to the inhibition of the reproductive axis. In the same line, we have demonstrated that AMPK signaling in Kiss1 neurons has a discernible role in the metabolic control of puberty, since conditional elimination of the AMPKα1 subunit from Kiss1 neurons protected pubertal female mice from the delay in the onset of puberty induced by post-weaning subnutrition (48). In good agreement, our data in immature female rats documented that pharmacological activation of central AMPK suppressed *Kiss1* expression in the ARC, together with its action in terms of induction of delayed puberty (48).

Interestingly, the activity of AMPK in the metabolic modulation of puberty and the reproductive axis is not restricted to its action in Kiss1 neurons, but also involves regulatory effects in GnRH neurons. Thus, conditional ablation of AMPKα1 from GnRH neurons resulted in accelerated puberty and enhanced responses to exogenous kisspeptin, both in pubertal and adult mice, suggesting that, in normal conditions, AMPK is also suppressing GnRH activity (51). In addition, mice lacking AMPK activity in GnRH neurons also displayed partial resistance to the suppressive impact of energy deprivation on the gonadotropic axis (51). Of note, the nutritional control of GnRH neurons also involves the regulatory actions of the G protein-coupled-receptor kinase-2, GRK2, as putative negative modulator of kisspeptin receptor in situations of subnutrition (52). Thus, conditions of energy deficit can suppress GnRH neurons via AMPK- and GRK2-dependent mechanisms.

### 3.2 mTOR and the metabolic control of puberty and reproduction

As a putative signal for energy abundance, positively modulated by leptin, the eventual role of mTOR signaling in the hypothalamus in the metabolic control of puberty was explored by our group over a decade ago (53). Indeed, our experimental data in female rats documented that intact brain signaling via mTOR is mandatory for normal pubertal progression. Moreover, pharmacological blockade of central mTOR by rapamycin not only suppressed ARC expression of *Kiss1*, which is a major puberty-promoting signal, but prevented also the permissive effects of leptin on puberty onset. Thus, in immature female rats subjected to chronic subnutrition, the pubertal delay induced by the state of negative energy balance could be rescued by simultaneous treatment with leptin, but this permissive response was abrogated by co-administration of rapamycin, as a means to inhibit mTOR activity (54). Admittedly, however, it remains unsolved if mTOR conducts such permissive/positive effect directly in Kiss1 neurons and/or operates in upstream afferents to Kiss1 cells. Notwithstanding, this evidence points to a major role of brain mTOR signaling in the metabolic control of puberty.

In this context, the function of several up-stream regulators of the mTOR pathway in the control of Kiss1 neurons has been more recently explored using functional genomics. Conditional ablation of the catalytic subunit of PI3K in Kiss1 neurons has been shown to reduce ARC kisspeptin content and to suppress reproductive function in mice, predominantly in females (55). Importantly, PI3K has been proposed as an integratory hub for conveying the actions of metabolic hormones with key roles in reproductive function, such as leptin. More recently, the function of PTEN (for *phosphatase and tensin homolog*) has been studied in Kiss1 neurons using also genetic ablation approaches; PTEN is known to inhibit PI3K activity. Thus, conditional elimination of PTEN from Kiss1 neurons caused hypertrophy of Kiss1 neurons and enhanced kisspeptin fiber density in mice, which was associated with exaggerated mTOR activity, especially in females (56). From a functional standpoint, these responses were linked to a situation of refractoriness to the negative impact of fasting on luteinizing hormone (LH), as main hormonal signal for gonadal stimulation. These findings are compatible with a function of PTEN to inhibit the PI3K/mTOR pathway in Kiss1 neurons, whereby it can contribute to the suppression of the reproductive axis in situations of nutritional deprivation.

### 3.3 SIRT1 and the metabolic control of puberty and reproduction

In line with its putative role as co-regulator of AMPK and fuel-energy sensor at the hypothalamus, SIRT1 has been documented to participate in the control of central components of the reproductive axis. The first evidence for such a reproductive dimension came from observations in Sirt1 null mice, which documented a state of hypogonadotropic hypogonadism, due to impaired migration of GnRH neurons (57), that renders Sirt1 KO mice infertile (58), due to a dramatic reduction in the number of GnRH neurons (57) and in the levels of circulating gonadotropins (59). This function of SIRT1, however, seems to be engaged in early developmental events, rather than dynamic regulatory actions linked to fluctuations in the whole-body metabolic status. The latter, however, has been documented by the demonstration of a relevant role of hypothalamic SIRT1, particularly in ARC Kiss1 neurons, in the regulation of puberty and its modulation by nutritional cues.

Expression analyses in immature female rats documented that hypothalamic SIRT1 protein levels decrease, whereas *Kiss1* expression increases during the transition between the infantile and pubertal period. Hypothalamic SIRT1 levels increased in models of pubertal undernutrition, bound to pubertal delay, whereas conditions of early obesity, linked to accelerated puberty, were associated with decreased SIRT1 content in the hypothalamus (60). Interestingly, SIRT1 protein content in Kiss1 neurons mirrored these profiles in the models of subnutrition (with increased SIRT1 levels) and obesity (with decreased SIRT1 content). In good agreement, enhanced SIRT1 activity, caused either by a pharmacological agent or virogenetic over-expression in the ARC, delayed puberty onset in female rats; a response that was associated with a suppression of *Kiss1* expression after pharmacological activation of SIRT1. Overall, this evidence suggests that SIRT1 actually operates as a repressor of *Kiss1*, which is modulated by the nutritional status, and can transduce part of the modulatory effects of either over- or undernutrition on pubertal timing.

The molecular substrate for the regulatory effects of SIRT1 on puberty is likely connected with its capacity to epigenetically modify the chromatin landscape of the *Kiss1* promoter, mainly in the ARC. Thus, during the normal pubertal transition, SIRT1 is evicted from the *Kiss1* promoter, allowing also the removal of other epigenetic repressors, such as EED, therefore adopting a permissive chromatin configuration that enhances *Kiss1* transcription. In conditions of accelerated puberty due to early over-feeding, this eviction is advanced, leading to precious elevation of *Kiss1* expression. In contrast, in conditions of subnutrition, removal of SIRT1 from the *Kiss1* promoter is deferred and this locks the chromatin landscape in a repressive configuration that reduces *Kiss1* expression and, thereby, delays puberty (60). Therefore, SIRT1 represents a link between nutritional status and the epigenetic machinery regulating the *Kiss1* promoter and pubertal maturation. Of note, while no evidence has been presented for a similar role of hypothalamic SIRT1 in the nutritional control of adult reproductive function, our preliminary observations strongly suggest that SIRT1 signaling in Kiss1 neurons in the rostral hypothalamus in adult female rats, that play a prominent role in the control of ovulation, is also relevant for the suppression of the pre-ovulatory surge of gonadotropins, as major hormonal trigger of ovulation, in conditions of energy deficit. Finally, it is interesting to note that over-expression of a mutant form of SIRT1, devoid of deacetylase activity, in astrocytes lowered *Kiss1* expression and perturbed reproductive function, pointing out that SIRT1 signaling in astroglia might also regulate the Kiss1 system in mice (61). Whether, as is the case in energy homeostasis, other members of the SIRT family participate also in the control of the reproductive axis and its modulation by metabolic cues, is yet to be elucidated.

## 4 Role of lipid sensing mechanisms in the hypothalamic control of energy homeostasis

Besides the role of hypothalamic energy sensors in the control of metabolic homeostasis and the nutritional modulation of puberty and fertility, emerging evidence supports that relevant hypothalamic metabolic pathways, including those governing feeding and body weight, may sense lipid nutrients and lipid mediators, driving also adaptive responses to maintain energy homeostasis. This phenomenon is termed hypothalamic “lipid sensing”, whose molecular mechanisms and interactive players are yet to be fully disclosed (62). In this context, there is a growing body of evidence supporting that fatty acid (FA) sensing in hypothalamic neurons provides signals regarding the metabolic state of the body, therefore enabling precise adjustments for whole-body energy homeostasis (63). Among the putative mechanisms involved, FA receptors have been recently recognized as relevant players in this phenomenon, since these are capable to bind and/or transduce the actions of FAs (64), likely providing an additional layer of sophistication to the brain systems for fine-tuning metabolism. In addition, other factors involved in lipid sensing, including transporters, nuclear receptors and lipid mediators, also contribute to the hypothalamic control of metabolic homeostasis, as briefly summarized in this section.

### 4.1 Hypothalamic FA receptors and the neuroendocrine control of metabolism

FAs are indispensable constituents of the plasma membrane and a highly efficient energy source. In addition, free FA (FFA) are of special relevance due to their capacity to operate as signaling molecules, with regulatory actions on gene expression and energy homeostasis at various physiological and pathological conditions (65). The regulatory actions of FFAs are mediated by signaling pathways initiated upon binding to FFA receptors (FFAR) present in the cell membrane (66). Different members of the FFAR family are abundantly expressed in multiple metabolic tissues and display ligand specificity by classes of FFA, therefore operating as genuine lipid sensors, able to trigger adaptive metabolic responses (65).

Among FFARs, receptors for long-chain fatty acids (LCFAs; with a chain length of 14-22 carbon atoms) have been shown as key players in the regulation of energy balance. Solid evidence supports that these receptors contribute to sense fluctuations of LCFA in the hypothalamus, with a prominent role in the regulation of whole-body energy metabolism (62, 67). In this context, FFAR4 (aka, GPR120), a membrane GPCR exclusively activated by LCFAs, is particularly relevant in the control of whole-body energy homeostasis (66). It is expressed in several metabolic tissues, including the brain, where FFAR4 has been detected in the hypothalamus, including the ARC (68). Central activation of FFAR has been shown to acutely decrease food intake (68), while administration of the LCFA, docosahexaenoic acid (DHA), prevented the inflammatory state induced by TNF-alpha in an hypothalamic cell line, rHypoE-7, that abundantly expresses FFAR4 (69). While these data may suggest that this FFAR may mediate the beneficial metabolic effects of omega-3 FAs, chronic administration of a FFAR4 agonist did not change energy expenditure or body weight in mice fed high-fat content diet (68).

Another important LCFA receptor is FFAR1 (aka, GPR40), which can be activated not only by LCFAs but also by medium-chain fatty acids (MCFAs, with a chain length of 6-14 carbons). FFAR1 participates in a wide range of physiological functions and its expression has been reported in several tissues, including the CNS. Of note, FFAR1 has been shown to be widely expressed in hypothalamic neurons directly involved in the control of energy homeostasis (64, 70). In this context, a recent study has documented that central pharmacological activation of FFAR1 in diet-induced obese mice decreased body weight and increased energy expenditure, while virogenetic knockdown of FFAR1 in ARC POMC neurons of obese mice evoked hyperphagia and body weight gain, as well as the development of hepatic insulin resistance and steatosis (70). These data highlight the relevance of hypothalamic FFAR1 signaling in the control of adult metabolic homeostasis.

Another MCFA receptor, GPR84, has been found to be expressed also in several metabolic tissues, including the brain (71), where its presence within the hypothalamus has been reported (64). While the information of the roles of GPR84 in the central control of metabolic homeostasis remains scarce, it has been shown that icv treatment with a GPR84 agonist reduced food intake in the rainbow trout together with an increase in hypothalamic mRNA levels of POMC and CART, and a decrease in NPY and AgRP levels (71). Further studies are needed to address the putative role of this FFAR as a hypothalamic lipid sensor in mammals.

Finally, FFARs sensitive to short-chain fatty acids (SCFAs; up to 6 carbon atoms), produced from the fermentation of dietary fibers by the gut microbiota (72), have been shown also to participate in the regulation of various physiological processes, including the maintenance of energy balance. These include FFAR2 (aka, GPR43) and FFAR3 (aka, GPR41), whose expression has been reported in several tissues, including the brain (65). In this context, FFAR2 and FFAR3 are expressed in the hypothalamus of rodents (73, 74). Although the exact mechanism whereby hypothalamic FFAR2/3 exert their metabolic actions is yet to be fully clarified, it has been shown that high fat diet exposure results in increased expression of FFAR3 in the PVN, associated with decreased butyrate levels, as putative contributing factor for development of inflammation and hypertension, while virogenetic silencing of FFAR3 in the PVN attenuated these adverse responses, i.e., tissue inflammation and hypertension, in rats (75).

### 4.2 Lipid transporters and nuclear receptors and the neuroendocrine control of metabolism

Besides FFARs, elements involved in the translocation of certain FAs inside the cell or the intracellular sensing of lipid species have been suggested to serve a role as lipid sensors themselves. Among these, CD36 (for *cluster of differentiation 36*) is a transmembrane protein expressed in various cell types, including hypothalamic neurons and astrocytes (76). CD36 is primarily known for its dual capacity as both facilitator of LCFAs transport and initiator of signaling cascades upon FA binding (77). Its role as hypothalamic lipid sensor is supported by its documented function as the main mediator of FFA actions in the VMH, as well as its prominent contribution to lipid sensing in glucosensing neurons in the ARC (76). Moreover, depletion of CD36 in the VMH/ARC region in rats fed on high-fat diet caused subcutaneous fat accumulation and increased leptin levels, together with insulin resistance, without an overall effect on food intake and body weight (78).

In addition, FA transport proteins (FATPs), a family of six transmembrane transporters (FATP1-FATP6) involved in the cellular uptake of FAs and their acylation (76), also contribute to mediating part of the actions of FFA to modulate metabolism, energy homeostasis, and lipid storage. Expression of FATP1 has been detected in neurons of the VMH (63), and *in vitro* studies strongly suggest that FATP1 substantially contributes to the brain uptake of lipid species, such as DHA and oleic acid (OA) (79, 80). Given the proven role of changes in OA and DHA upon hypothalamic neuronal populations, such as POMC and AgRP neurons, it is tenable to consider FATP1 as key component in central lipid sensing. Furthermore, FATP4 expression has been detected in neurons and astrocytes of the VMH in mice (63), and virogenetic silencing of FATP4 in the VMH increased body weight, food intake, fat mass and leptin levels in mice.

Once inside the cell, FAs can function as signals of energy status by acting on nuclear receptors, also involved in lipid sensing, which upon binding operate as transcriptional factors regulating the expression of genes involved in lipid metabolism and energy homeostasis (81). The most prominent example is the family of peroxisome proliferator-activated receptors (PPARs), ligand-activated nuclear receptors that play a crucial role in regulating essential physiological functions, such as glucose and lipid metabolism, as well as energy balance. Upon FA binding, PPARs are translocated to the nucleus and heterodimerize with another nuclear receptor, the retinoid X receptor (RXR), acting as transcription factors by binding to peroxisome proliferator response elements (PPREs) which allows the heterodimer to activate or repress transcription (64). Three different PPAR isoforms -PPARα, PPARβ/δ and PPARγ- have been identified, and their involvement in lipid metabolism has been well documented. While all PPAR isoforms have been detected in the CNS, their expression levels differ among the different isoforms, suggesting distinct roles in regulating metabolism and energy homeostasis. The role of PPARγ as lipid sensor seems to be especially relevant, as its expression has been reported in hypothalamic areas involved in energy homeostasis and feeding behavior (82), while PPARγ levels in AgRP/NPY neurons are sensitive to the nutritional state (83). Moreover, central overexpression of PPARγ in diet-induced obese mice resulted in decreased ghrelin and NPY mRNA levels, while POMC mRNA levels were increased (84). On the contrary, deletion of PPARγ in POMC neurons in high-fat diet fed mice attenuated hyperphagia, increased energy expenditure, and protected from obesity and leptin resistance (85). All these data attest to a prominent role of PPARγ in hypothalamic lipid sensing and metabolic control.

### 4.3 Hypothalamic lipid mediators and the neuroendocrine control of metabolism

In a broad sense, another component of the lipid sensing mechanisms involves lipid species, other than FFA, with signaling capacities and ability to modulate central pathways governing metabolism. A prominent example is hypothalamic ceramides, lipid molecules composed of a sphingosine moiety bound to FA with a range of chain length, from 14 to 30 carbon atoms (86). Besides the proven role of ceramide accumulation in peripheral tissues in the pathophysiological control of metabolism, compelling evidence, gathered in the last decade, has documented a relevant role for hypothalamic ceramides, in connection with ER stress responses, in mediating the impact of adverse metabolic conditions, such as obesity, at the whole-body level. In fact, ceramide accumulation in the VMH, coupled to ER stress, has been shown to decrease thermogenesis and promote body weight gain and insulin resistance (87). Furthermore, hypothalamic ceramide signaling participates in mediating the metabolic actions of key hormonal regulators, including leptin, ghrelin and estrogens; for instance, the capacity of leptin and estrogens to decrease the hypothalamic levels of ceramides has proven relevant for mediating their effects in terms of induction of thermogenesis and reduction of body weight (87, 88). On the other hand, the pathogenic role of central ceramide accumulation in promoting metabolic perturbations is documented by the fact that inhibition of ceramide synthesis in the brain can ameliorate insulin resistance in leptin-receptor mutant Zucker rats (89), while blockade of ceramide synthesis in the VMH can improve the metabolic profile in rat models of obesity (87). Similarly, CerS6-mediated ceramide synthesis in hypothalamic neurons, as those expressing POMC or SF1, likely contributes to the metabolic deregulation induced by obesogenic diets (90), while ceramides participate also in other relevant brain functions, such as myelination (91).

Finally, bile acids (BA), as derivatives of the lipid precursor, cholesterol, synthesized in the liver (primary BA) and later transformed into secondary BA by the gut microbiota, have gained momentum as putative metabolic mediators, acting in part via hypothalamic circuits (92). Initially considered merely as detergent molecules essential for intestinal lipid absorption, BAs are known to operate via specific nuclear and cell-surface receptors to conduct a variety of regulatory effects (93). Indeed, BAs have been detected in the brain at a concentration that correlates with their circulating levels (94), and BAs have been shown to activate their receptors also in the hypothalamus (95), therefore supporting a role of BA signaling in the mechanisms for central lipid sensing.

Indeed, recent studies have demonstrated that the BA-selective, G protein-coupled receptor, TGR5, operates at the hypothalamus to convey part of the metabolic actions of BAs. Thus, hypothalamic BA content is decreased in obese mice and virogenetic-mediated suppression of TGR5 in the mediobasal hypothalamus promoted obesity development. Conversely, central activation of TGR5 reduced body weight (95). Moreover, specific deletion of TGR5 in AgRP neurons of mice has been recently shown to increase in food intake (96), pointing to the contribution of this hypothalamic pathway for the anorectic effects of BAs.

## 5 Role of lipid sensing mechanisms in the hypothalamic control of reproduction

While our knowledge of the role of hypothalamic lipid sensing in the control of whole-body homeostasis has substantially expanded during the last decade, whether analogous mechanisms operate in the metabolic regulation of puberty and fertility remains largely unexplored. From a conceptual standpoint, such intersection between central lipid sensing and the centers governing the reproductive axis is tenable, given the sensitivity of the pathways controlling puberty and fertility to nutritional and metabolic cues, and the proven interplay between reproductive- and metabolic-regulatory circuits within the hypothalamus. Indeed, emerging evidence suggests a role of specific lipid mediators in the perturbations of puberty in conditions of early-obesity (97), while hypothalamic PPARγ seems to modulate part of reproductive responses to high-fat diet in mice (82). Yet, the putative “reproductive” roles of other components of the hypothalamic lipid sensing mechanisms have not been studied yet.

Our group has provided evidence for a novel brain pathway, involving *de novo* ceramide synthesis at the PVN, in the metabolic control of puberty and its alterations after early-onset obesity in rats. Thus, as it is the case in adulthood, the hypothalamic ceramide content was increased in pubertal female rats subjected to postnatal overfeeding, displaying early obesity, which was associated with accelerated puberty. Activation of central *de novo* ceramide synthesis in immature female, but not male rats, also resulted in advancement of the age of puberty onset despite no changes in body weight, whereas blockade of brain *de novo* ceramide synthesis resulted in delayed puberty and obliterated the stimulatory effects of kisspeptins on pubertal maturation. Notably, this phenomenon was conducted via gonadotropin-independent modulation, and involved a previously unnoticed hypothalamic pathway, involving the PVN and the sympathetic innervation of the ovary. In fact, obese pubertal female rats were shown to display changes in kisspeptin innervation of the PVN and increased content in this nucleus of the enzyme, serine palmitoyltransferase long chain base subunit 1 (SPTLC1), responsible for the initial, limiting step in *de novo* ceramide synthesis (97). In fact, virogenetic blockade of SPTLC1 in the PVN of obese female rats largely prevented pubertal acceleration due to overweight. These findings support that ceramide-related pathways are key to central alteration of female puberty in conditions of early obesity.

In addition, functional genomic analyses have evaluated the role of another component of lipid sensing, namely hypothalamic PPARγ signaling, in the control of puberty and reproduction. Thus, female mice engineered to lack PPARγ in mature neurons displayed normal age of pubertal maturation but markers of ovulatory dysfunction, with smaller litters and a reduction in the number of oocytes released per ovulation. In addition, neuron-specific PPARγ KO females displayed alterations in ovarian cycle length and LH levels, as well as hemorrhagic corpora lutea in the ovaries; yet, they were protected from obesity-induced leptin resistance and ovarian cycle irregularities (82). Of interest, neuronal ablation of PPARγ did not alter body weight or glucose/insulin homeostasis in this genetic model. Whether other means of manipulation (e.g., pharmacological) of brain PPARγ signaling may influence reproductive function is yet to be defined. Likewise, whether other lipid sensing mechanisms in the hypothalamus, such as those mediated by FFAR, FA transporters or the BA receptor, TGR5, participate in the control of puberty and reproduction has not been thoroughly addressed and warrant future investigation. Yet, our preliminary observations suggest FFARs, such as GPR84, and PPARγ might participate in the hypothalamic control of puberty, with a variable role depending on the maturational stage and metabolic status. In the same vein, a very recent study has pointed out a putative role of TGR5 in the central regulation of puberty onset in female rats (98). It must be noted that, in addition, PPARγ signaling at the pituitary, the placenta and the ovary has been shown to participate in the control of female reproduction (99), and the use of PPARγ agonists has been proposed in the context of reproductive disorders bound to metabolic alterations, such as polycystic ovary syndrome.

## 6 Conclusions

In recent years, we have witnessed a substantial expansion of our knowledge of the hypothalamic mechanisms governing whole-body metabolism and reproduction, and particularly of the central pathways responsible for the metabolic control of puberty and fertility. In this context, besides the elucidation of the important reproductive roles of key peripheral hormones, such as leptin, insulin and ghrelin, and central transmitters with pivotal functions in energy homeostasis, recognition of fundamental components of the reproductive brain, such as Kiss1 neurons, sensitive to nutritional and metabolic cues, have paved the way for the integral comprehension of the neuroendocrine mechanisms whereby metabolism and reproduction are tightly connected, and eventually deregulated in adverse conditions.

On top of such neuropeptide framework, it has become evident recently that additional layers of molecular mediators participate in the fine tuning of puberty and reproductive function, and their modulation by metabolic signals. As a clear example, during the last decade it has been disclosed that epigenetic regulatory mechanisms operate to precisely control pubertal maturation and to convey the influence of the nutritional cues on puberty onset (100). In the same vein, the role of different cellular energy sensors, such as AMPK, mTOR and SIRT1, acting on hypothalamic circuits converging on Kiss1 and GnRH neurons, have been shown to participate in the metabolic control of puberty, coupling body nutritional and metabolic status to pubertal maturation and, eventually, fertility. In addition, illuminated by recent evidence pointing out that lipid sensing mechanisms are key components for the hypothalamic regulation of whole-body metabolism (62), the roles of elements of such lipid sensing pathways in the control of reproductive function have begun to be explored recently. These include, but are not restricted to, the roles of hypothalamic ceramides in obesity-induced pubertal acceleration in females, as well as the role of hypothalamic PPARγ in the modulation of the female reproductive axis and its perturbation by obesogenic diets. As important note, it must be stressed that most of our understanding of roles of these energy-sensing mechanisms and lipid mediators in the control of puberty, as key maturational event of the reproductive axis highly sensitive to metabolic cues, derives from studies in female rodents, whereas our knowledge of the eventual function of these signaling pathways in males remains virtually null. Given that puberty and reproductive function in the male are also sensitive, albeit to a lesser extent, to body energy status, further research is warranted to analyze whether sex differences exist regarding the pathways for the integrative control of energy balance and reproduction; a contention supported by our recent evidence for the female-specific role of hypothalamic ceramide signaling in the control of puberty onset, which is not observed in male rats (97).

In addition, a major challenge that remains to be solved is how the proposed mechanisms for the integral regulation of whole-body metabolism and reproductive function actually integrate within the complex framework defined by the multiple neuronal and glial populations of the hypothalamus. Recent single cell RNA sequencing studies in the mouse hypothalamus, comparing opposite nutritional states (feeding ad-libitum vs. acute fasting), have revealed significant transcriptional changes in AgRP neurons and other cell types, activated during food deprivation (11). Among the differentially-expressed genes, *Zbtb16*, encoding a transcription factor with a relevant role in neurogenesis; *Fam107b*, that codes for a stress response mediator; *Vgf*, a neuropeptide precursor involved in energy homeostasis; and *Sv2c*, a glycoprotein involved in neurotransmitter release, have been highlighted (11). Whether these factors can contribute to the metabolic control of the reproductive axis is yet to be defined, but the *Vgf*-encoded neuropeptide, TLQP-21, has been previously shown to modulate puberty onset and reproductive hormone secretion in rats (101). Importantly, the above RNA-seq studies not only aid in identifying novel and essential components within the different hypothalamic cell populations that control organism functions, including metabolism and reproduction, but also pave the way for comprehensive approaches involving various multi-omics strategies and specific manipulations, targeting different cell types (e.g., cell type-specific viral transfections, as well as chemo- and optogenetic manipulations). These approaches will allow the generation of functional connectivity mappings among different hypothalamic cell types responsible for the integral control of energy balance and reproduction.

Finally, it is anticipated that future efforts in this domain will aim to elucidate the complete set of biological actions and mechanisms of such metabolic and lipid sensing pathways. The resulting knowledge will be instrumental not only to gain deeper insight into the physiological mechanisms governing puberty and fertility, but also to identify novel therapeutic targets for improved management of high prevalent metabolic and reproductive conditions, ranging from pubertal disorders to polycystic ovary syndrome and obesity.
